# Method for Emotion Recognition of EEG Signals Based on Recursive Graph and Spatiotemporal Attention Mechanism

**DOI:** 10.3390/brainsci16040377

**Published:** 2026-03-30

**Authors:** Dong Huang, Lin Xu, Yuwen Li

**Affiliations:** 1 School of Instrument Science and Engineering, Southeast University, Nanjing 210096, China; 220233676@seu.edu.cn; 2 National Key Laboratory of Information Systems Engineering, Nanjing 210096, China; xulin19851116@sina.com

**Keywords:** EEG, recurrence plot, TCSA, efficientnet, DEAP, DREAMER, emotion

## Abstract

Emotion recognition plays a crucial role in human–computer interaction and mental health applications. Traditional Electroencephalogram (EEG)-based emotion recognition methods are limited in classification accuracy due to their neglect of the spatiotemporal characteristics of the signals and individual differences. This study proposes a novel EEG emotion recognition framework that integrates spatiotemporal features to enhance performance through the following innovations: (1) the use of a Recurrence Plot (RP) to transform one-dimensional EEG signals into two-dimensional images, enhancing the representation of nonlinear dynamic features; (2) the design of a Spatiotemporal Channel Attention Module (TCSA), which combines temporal convolution, channel, and spatial attention mechanisms to optimize the capture of complex patterns; and (3) the integration of the lightweight and efficient network Efficientnet to construct the TCSA-Efficientnet classification model. On the Database for Emotion Analysis using Physiological Signals (DEAP) dataset, the proposed method achieves accuracy rates of 99.11% and 99.33% for valence and arousal classification tasks, respectively. On the Database for Emotion Recognition Using EEG and Physiological Signals (DREAMER) dataset, the method achieves accuracy rates of 98.08% and 97.49%, outperforming other EEG-based emotion classification models on both datasets. This demonstrates its advantages in accuracy, robustness, and generalization.

## 1. Introduction

Emotion is an indispensable factor in human life, profoundly influencing decision-making, social behaviors, and mental health. It not only is a subjective response of an individual to external stimuli but also affects cognitive functions, emotional judgments, and behavioral decisions. Therefore, emotion plays a significant role in daily life, especially in complex decision-making, emotional regulation, and interpersonal communication. In recent years, the potential applications of emotion recognition technology have gradually emerged across various fields, particularly in mental health treatment, human–computer interaction, and smart healthcare. Emotion recognition technology not only helps improve therapeutic outcomes for patients but also enhances the intelligence and personalization of human–computer interactions.

Emotion recognition technology is primarily based on two types of signals: physiological and non-physiological signals. Non-physiological signals, such as facial expressions and speech intonation, are easy to collect and widely applied; however, they are often influenced by subjective emotional fluctuations, environmental factors, and social behaviors. In contrast, physiological signals, such as electroencephalogram (EEG) [1], electrocardiogram (ECG) [2], galvanic skin response (GSR) [3], and heart rate variability (HRV) [4], can more objectively and accurately reflect emotional states. These signals are directly related to the body’s physiological responses and are less susceptible to external subjective factors, thus providing higher stability and consistency. Consequently, physiological signals offer new possibilities for emotion monitoring, especially for patient groups unable to engage in verbal communication or facial expressions.

Among physiological signals, EEG records brain activity through the summation of postsynaptic potentials from synchronized pyramidal cells, reflecting the brain’s electrophysiological activity. Since EEG can monitor emotional states in real-time, it has gained widespread attention in emotion recognition research. To understand the current landscape, we review the evolution of EEG analysis methods, ranging from traditional machine learning to deep learning approaches.

Early EEG emotion recognition research primarily relied on traditional machine learning methods, which generally involve two steps: feature extraction and classification. In the feature extraction phase, researchers extract time-domain, frequency-domain, and time–frequency-domain features. For instance, Nawaz et al. [5] systematically compared statistical features, fractal dimension (FD), higher-order spectra (HOS), and wavelet features. Zheng et al. [6] proposed a method using deep belief networks to automatically extract features, validated on several public datasets. Petrantonakis and Hadjileontiadis [7] introduced a feature extraction method based on higher-order cross-spectral analysis combined with support vector machines (SVM). Similarly, Lin et al. [8] used power spectral density (PSD) combined with SVM to classify four basic emotions. Additionally, k-nearest neighbors (k-NN) and random forests have been widely applied. Koelstra et al. [9] compared various algorithms on the Database for Emotion Analysis using the Physiological signals (DEAP) dataset and found that the random forest model based on time–frequency features performed exceptionally well. With the development of techniques, researchers explored more complex methods. Jenke et al. [10] proposed a sparse representation-based method combined with linear discriminant analysis (LDA). Wavelet transform is also widely used [11]; Li et al. applied discrete wavelet transform to divide EEG signals into frequency bands, calculating entropy and energy for KNN classification [12]. Kernel-based SVMs [13] and Hidden Markov Models (HMM) [14] have also been introduced to capture nonlinear and temporal dynamic characteristics.

In recent years, deep learning methods have made significant advancements, automatically extracting features from raw EEG signals and avoiding cumbersome manual design. For example, Li et al. [15] proposed a convolutional neural network (CNN) model that learns spatiotemporal features directly from raw signals. Li et al. [16] introduced a spatial frequency convolution self-attention network (SFCSAN) to obtain frequency information across bands. Moreover, deep learning models based on attention mechanisms have gained increasing attention. For instance, Zhu et al. [17] proposed TAGAT, a graph attention-based method that leverages the spatial structure of EEG channels. Meanwhile, Generative Adversarial Networks (GANs) have been introduced for data augmentation; Luo et al. [18] proposed a GAN-based method that generates high-quality EEG data, significantly improving classification performance. Combining image processing techniques with attention mechanisms has also created new opportunities. Converting EEG signals into images preserves temporal and spatial information, while attention mechanisms dynamically adjust the focus on important signal regions, improving accuracy and efficiency.

In addition to single-modal signals, multimodal emotion recognition methods have attracted increasing interest. These methods typically combine EEG with other physiological signals (ECG, GSR) or external data (facial expressions, speech). For example, Soleymani et al. [19] proposed a framework combining EEG, ECG, and GSR, significantly enhancing performance. Similarly, Zhang et al. [20] pre-processed speech, video, and text, using deep learning to extract features and performing information fusion. Additionally, Lan et al. [21] introduced deep generalized canonical correlation analysis with an attention mechanism to implement multimodal adaptive fusion.

Despite these significant advancements, several challenges remain. Many existing methods focus only on global graph structures, neglecting the importance of local spatial information. Furthermore, emotion intensity changes over time, and using only a single time segment may result in the loss of temporal information. Therefore, it is crucial to consider the relationships across the overall time period to improve recognition accuracy and efficiency.

**Figure 1:**
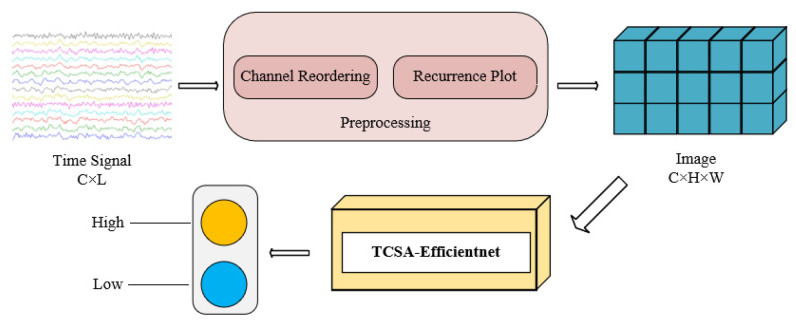
The overall framework of the proposed EEG emotion recognition method. The colored lines in the input time signal represent multi-channel EEG signals collected from different scalp electrodes (or filtered into different frequency bands, e.g., delta, theta, alpha, beta, gamma). The framework consists of three key stages: preprocessing (including channel reordering and recurrence plot transformation to convert 1D EEG time series into 2D images), feature extraction and classification via the TCSA-Efficientnet model, and final emotion recognition output (high/low valence/arousal). The main contributions of this paper can be summarized as follows. An overview of the entire proposed framework is illustrated in Figure 1.

This study proposes an EEG emotion recognition method based on image processing, as illustrated in Figure 1. Traditional EEG signals are transformed into images using a recursive graph algorithm, which calculates the similarity between different time points in a time series and considers the relationships between overall time periods. The framework also integrates the Efficientnet [22] model and the designed Temporal Convolution with Spatial and Channel Attention (TCSA) module to fully explore the complex characteristics of EEG signals. This approach aims to enhance the accuracy, robustness, and real-time performance of emotion recognition, advancing the application of emotion recognition technology in fields like mental health and intelligent healthcare.

1. Recurrence Plot-Based Nonlinear Feature Representation for EEG:

Recurrence plots (RP) are adopted to convert one-dimensional EEG signals into two-dimensional visual representations, which effectively preserve the nonlinear dynamic characteristics of EEG signals and provide a suitable input format for image-based recognition models.

2. TCSA Mechanism: The proposed TCSA mechanism integrates temporal convolution with spatial-channel attention, optimizing the model’s focus on key spatiotemporal features in EEG signals, thus improving emotion recognition accuracy.

3. Integration of TCSA with Efficientnet:

Integrating TCSA with the Efficientnet model leverages its efficiency and the spatiotemporal feature extraction capabilities of TCSA, further enhancing the performance and robustness of emotion classification tasks.

## 2. Materials and Methods

### 2.1. Methodology

In this section, the data preprocessing techniques applied to the raw EEG signals are introduced. The key step in preprocessing is the transformation of the time-series signals into image format. This conversion helps capture the spatial and temporal dependencies within the signals, providing more effective input for subsequent deep learning models. Next, a convolutional module called TCSA is designed, which incorporates a time dimension based on the channel and spatial attention mechanisms. This is then combined with the Efficientnet classification model to improve the accuracy and computational efficiency of emotion recognition. Efficientnet is an efficient and lightweight deep learning architecture that achieves exceptional performance with fewer parameters through a carefully designed network structure.

#### 2.1.1. Preprocessing

**Figure 2:**
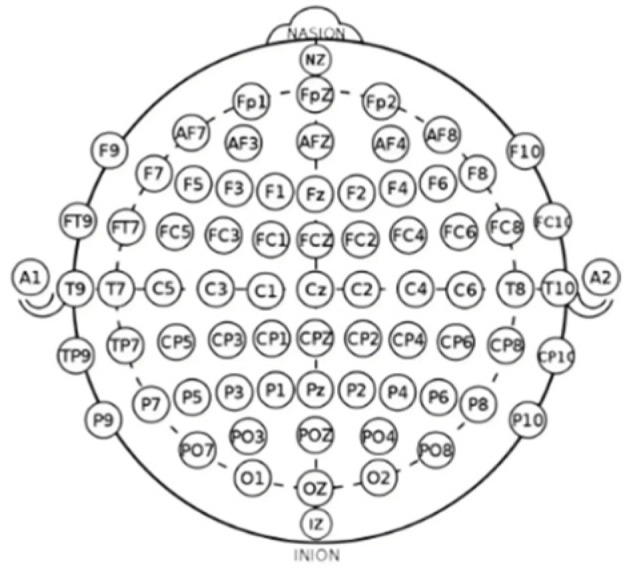
Electrode placement map of the International 10–20 system.

In this study, the EEG data of each participant underwent preprocessing, with the first step being the reordering of the original EEG channel sequence. This operation aimed to enhance the spatial characteristics of the signal, thereby improving the model’s ability to understand EEG signals. The original channel layout did not sufficiently account for the actual spatial distribution of different brain regions, which could limit the full utilization of spatial features in subsequent analyses. To better reflect the brain’s anatomical structure and functional region distribution, the reordering of the channels was based on the locations of different brain regions. The defined sequence drew upon the International 10–20 System [23] and incorporated the functional partitioning of the cerebral cortex, as illustrated in Figure 2, adjusting the channel sequence to more closely group electrodes corresponding to adjacent or functionally similar brain areas. This adjustment not only adheres to the functional partitioning of the cortical areas but also helps maintain local consistency within regions, thereby improving the deep learning model’s ability to capture spatial features.

After spatially reordering the EEG channels, we apply the RP (Recurrence Plot) [24] algorithm to transform each signal segment. The RP algorithm reveals the self-similarity and nonlinear characteristics within time series signals, effectively converting one-dimensional signals into two-dimensional images. Specifically, the RP algorithm constructs a recurrence matrix that describes the self-similarity in the time series. Let $x_{i}$ and $x_{j}$ represent the *i*-th and *j*-th data points in the time series, respectively, and the distance between them is calculated using the Euclidean distance metric:

(1) $$d\left(i,j\right)=\left\|x_{i}-x_{j}\right\|$$

In this process, $\left\|x_{i}-x_{j}\right\|$ represents the Euclidean distance between the *i*-th and *j*-th data points in the time series. In the RP algorithm, a threshold ε is used to determine whether two time points are considered similar. If $d\left(i,j\right)$ is smaller than the set threshold ε, the two points are regarded as similar, and the corresponding position in the recurrence matrix is assigned a value of 1; otherwise, it is assigned a value of 0. The formula for the recurrence matrix is as follows:

(2) $$R\left(i,j\right)=\theta \left(\varepsilon -\left\|x_{i}-x_{j}\right\|\right)$$

where θ(·) is the Heaviside step function, indicating that when $d\left(i,j\right)<\varepsilon$, $R\left(i,j\right)=1$; otherwise, $R\left(i,j\right)=0$.

Through this process, the resulting recurrence matrix can be considered as the “image” representation of the signal segment, revealing the temporal structure and complexity of the signal.

**Figure 3:**
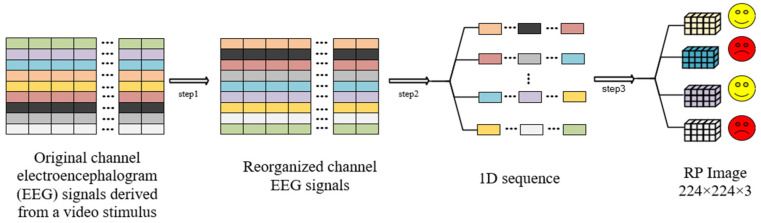
The EEG channels are spatially reordered according to brain regions and then flattened across channels into a one-dimensional time series. The signal is segmented into subsequences using a sliding window approach. After performing an arctangent transformation, the Recurrence Plot algorithm is applied to generate 224 × 224 images. Different colored blocks in the figure represent EEG signals from distinct scalp electrodes or functional brain regions, which are spatially reordered to group functionally adjacent brain areas in the preprocessing stage. The yellow smiley faces represent positive emotion states (e.g., high valence/high arousal), and the red frowny faces represent negative emotion states (e.g., low valence/low arousal), corresponding to the final emotion classification results output by the model.

To achieve this transformation, a sliding window approach is employed for the windowing process of the reconstructed one-dimensional EEG time series, as illustrated in the overall workflow in Figure 3. Considering that all datasets adopted in this study have a sampling rate of 128 Hz, the window length is set to 7680 sampling points (corresponding to a duration of 60 s), and the stride (step size) is set to 1920 sampling points (corresponding to a duration of 15 s, with the stride accounting for 25% of the window length). This configuration divides each signal segment into multiple consecutive and partially overlapping subsequences. Such parameter settings not only avoid temporal misalignment and boundary effects caused by approximate values of window length or stride in traditional segmentation methods but also ensure the continuity of temporal features through the 25% overlap ratio (15 s stride). For each subsequence, an arctangent transformation was first applied to suppress the interference of extreme values in the EEG signal on subsequent processing. Subsequently, the Recurrence Plot (RP) algorithm was utilized to convert the one-dimensional subsequence into a two-dimensional recurrence matrix: specifically, the one-dimensional EEG subsequence with a length of 7680 sampling points was input into the RP algorithm, which constructed a 7680 × 7680 square recurrence matrix. To match the input size requirement of the subsequent classification model, the 7680 × 7680 recurrence matrix was resampled to a 224 × 224 pixel dimension through bilinear interpolation during visualization. The single-channel 224 × 224 recurrence matrix is normalized to the intensity range of 0–255. Each scalar value in the matrix is then converted into a corresponding triplet of red, green, and blue (RGB) color components via pseudo-color mapping with the rainbow colormap. In this manner, the one-channel recurrence matrix is transformed into a standard 224 × 224 × 3 three-channel RGB image, forming a pseudo-color RP image. Ultimately, each original signal segment was transformed into this standardized three-channel RGB image format, which served as the input data for subsequent classification models. This approach not only preserves the spatiotemporal characteristics of EEG signals by converting one-dimensional time series into two-dimensional image format but also enhances the discriminability of nonlinear dynamic features through color-coded representation, contributing to improved accuracy and robustness in emotion classification tasks.

#### 2.1.2. TCSA

The spatial dimensions of the RP-based image correspond to the physical and temporal structure of EEG signals (e.g., electrode distribution and time steps), rather than being random pixel arrangements. Spatial attention is therefore introduced to learn the importance weights of different spatial locations, so as to enhance features from emotion-related brain regions and time steps while suppressing irrelevant ones. Channel attention is adopted to model the contribution of different feature channels, adapting to the three-channel RGB representation generated by pseudo-color mapping. Furthermore, temporal convolution is employed to capture dynamic patterns across time, since the RP is naturally constructed by measuring similarity across time points and is sensitive to temporal evolution.

**Figure 4:**
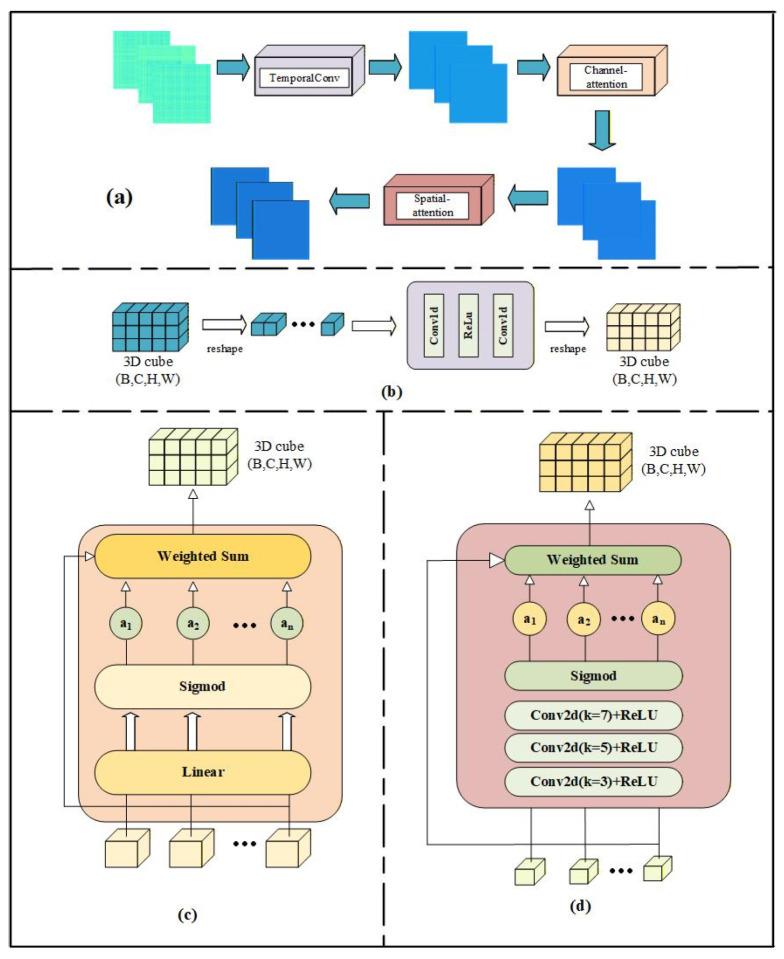
Overall architecture of the proposed TCSA module. (**a**) The overall framework of the TCSA module, which integrates three parallel branches for spatiotemporal feature extraction from EEG signals; (**b**) The Temporal Convolution branch, designed to capture dynamic evolution in the Recurrence Plot (RP) images; (**c**) The Channel Attention branch, which recalibrates feature importance across different channels; (**d**) The Spatial Attention branch, which localizes emotion-related brain regions and time steps. The arrows in the figure indicate the flow of data and features through each computational layer of the module. The fusion of these branches enables the extraction of robust spatiotemporal features from EEG signals. In EEG signals, emotional states are often reflected by pattern variations over long time spans rather than only local fluctuations. Meanwhile, the high individual variability and non-stationarity of EEG lead to unstable representations at different time scales. Traditional attention mechanisms focus mainly on local image features, which are insufficient to capture the long-range temporal dependencies and structured spatial information in the recurrence plot (RP). Different colored blocks in the figure represent feature maps at different processing stages (cyan for input features, blue for output features after network layer processing), and different colored boxes denote distinct computational layers.

To this end, we design the TCSA module, which embeds temporal convolution into a dual spatial and channel attention framework. The detailed architecture of the TCSA module is illustrated in Figure 4. Specifically, TCSA introduces convolutional operations along the temporal dimension, enabling the model to focus on discovering patterns at specific time scales. This helps capture the temporal characteristics within the RP, thereby enhancing the model’s ability to understand the input data and improving its generalization performance.

The TCSA module, built upon a combination of channel and spatial attention mechanisms, incorporates convolution along the temporal dimension. This is intended to strengthen the model’s ability to learn EEG signal features, particularly in capturing the temporal dynamics within RP.

The TCSA module first processes the input feature map through a Temporal Convolution module. Let $X\;\epsilon {\;R}^{C\times H\times W}$ (where C is the number of channels, and H and W represent the height and width of the feature map, respectively). In the Temporal Convolution module, the input feature map is rearranged into a tensor of shape [b·h, c, w], where each height dimension H is treated as a time step, and each time step contains W feature points. Subsequently, two 1D convolution layers are applied to extract features along the temporal dimension, and the output is restored to the original shape [b, c, h, w]. This operation helps capture the dynamic patterns in the RP that evolve over time. The feature map is then processed through the Channel-attention module. Let $X\;\epsilon \;R^{C\times H\times W}$ be reshaped into a matrix of size C×H×W, where the transformed feature map can be viewed as the features extracted from each channel along the spatial dimensions H and W. The flattened feature map $X_{permute}\in R^{C\times H\times w}$ is then passed through two fully connected layers. The first step is to compute:

(3) $$z_{1}=x_{permute}\times W_{1}+b_{1}$$

where W1 ϵ RC×Cr is the learned weight matrix, and $b_{1}$ is the bias term.

The intermediate value $z_{2}$ is computed by applying the ReLU activation function:

(4) $$z_{2}=\sigma \left(W_{2}\times ReLu\left(z_{1}\right)+b_{2}\right)$$

where W2 ϵ  RC×Cr is another learned weight matrix, $b_{2}$ is the bias term, *r* is the channel compression factor, and σ denotes the Sigmoid activation function. The channel attention weights computed are then multiplied with the input feature map to produce the weighted feature map $X_{att\_channel}$:

(5) $$X_{att\_channel}=X\times z_{2}$$

Subsequently, the weighted feature map undergoes processing through the Spatial-attention module, which utilizes convolution operations to learn spatial location weights. The feature map $X_{att\_channel}$, output from the channel attention module, is subjected to two convolution operations. The first convolution outputs $\frac{c}{r}$ channels, while the second convolution outputs *C* channels. The spatial attention feature $A_{spatial}$ is computed as follows:

(6) $$A_{spatial}=\sigma \left(BN\left(Conv2d\left(ReLu\left(Conv2d\left(X_{att\_channel}\right)\right)\right)\right)\right)$$

Once the spatial attention weights are obtained, they are multiplied with the channel-attended feature map $X_{att\_channel}$, resulting in the final weighted output $X_{att\_fianl}$:

(7) $${{X_{att\_final}=X}_{att\_channel}\times A}_{spatial}$$

Thus, the final output of the TCSA module can be expressed as:

(8) $${Output=X}_{att\_final}$$

Through temporal convolutions and the dual channel and spatial attention mechanisms, the TCSA module is able to adaptively learn the relevant features within the input signal, helping the model focus on the most informative spatiotemporal locations in the EEG signals.

#### 2.1.3. MBConv-TCSA

**Figure 5:**
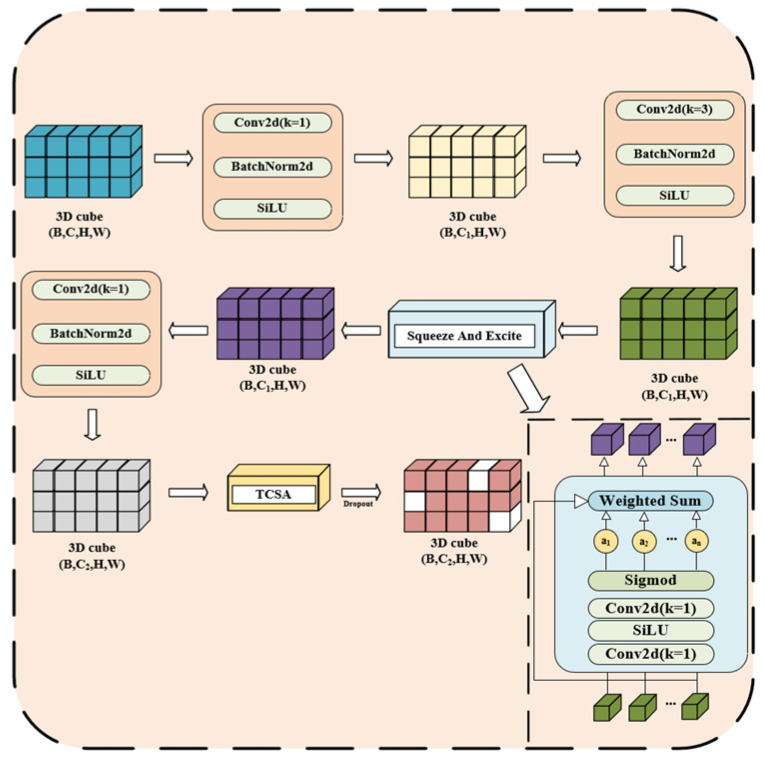
Structural diagram of the MBConv-TCSA module. The diagram illustrates the integration of the TCSA block within the Mobile Inverted Bottleneck Convolution (MBConv) architecture. It highlights the sequential flow from point-wise expansion and depthwise convolution to the dual-attention recalibration, designed to enhance spatiotemporal feature learning while maintaining computational efficiency. The arrows in the figure indicate the direction of data and feature flow through each computational layer. Different colored 3D cubes represent feature maps at distinct processing stages; the purple and green blocks denote input and output feature vectors for the TCSA attention module, respectively; and the yellow circular markers (a_1_, a_2_, …, a_n_) represent the learned channel/spatial attention weights generated by the module.

The proposed MBConv-TCSA (Mobile Inverted Bottleneck Convolution with TCSA) module is constructed by embedding the TCSA module into the standard Mobile Inverted Bottleneck Convolution (MBConv) block, as illustrated in Figure 5. This integration enables the model to effectively capture informative spatial and channel-wise features while maintaining high computational efficiency. The input tensor $x\in R^{C\times H\times w}$ first passes through a point-wise layer, which expands the number of channels from *C* to a larger size. The formula for the point-wise layer is as follows:

(9) $$x=\sigma \left(BN\left(Conv(x)\right)\right)$$

where Conv denotes the convolution operation, BN represents batch normalization, and σ is the SiLU activation function.

Next, the expanded features are processed through depthwise convolution. Depthwise convolution is a type of convolution where spatial convolutions are applied independently on each channel. The depthwise convolution operation is defined as:

(10) $$DWConv\left(x\right)=\sigma (BN(Conv(x)))$$

where Conv denotes the convolution operation, BN represents batch normalization, and σ is the SiLU activation function.

The key advantage of depthwise convolution lies in its ability to preserve spatial features while significantly reducing computational complexity. Specifically, it allows the model to learn spatial information independently on each channel, thereby avoiding the computational overhead of fully connected convolutions.

After the depthwise convolution, a Squeeze-and-Excite (SE) layer is introduced. The SE layer first performs global average pooling to obtain channel statistics z, and then adjusts the inter-channel weights through two convolutional layers to recalibrate the feature channels. This recalibration enhances important features while suppressing less important ones. The equations for the SE operation are as follows:

(11) $$z=\frac{1}{H\times W}\sum_{i=1}^{H}\sum_{j=1}^{W}Conv(x)$$

(12) $$s=\sigma (W_{2}\times SiLu\left(W_{1}\times z+b_{1}\right)+b_{2})$$

(13) $$x_{out}=x\times s$$

where $W_{1}\in R^{C*\frac{C}{r}}$ is the 1 × 1 convolution kernel of the first convolution layer, which performs channel compression by reducing the number of channels to $\frac{C}{r}$; $W_{2}\in R^{\frac{C}{r}*C}$ is the 1 × 1 convolution kernel of the second convolution layer, which performs channel restoration to recover the number of channels to C; $b_{1}{,\;b}_{2}$ are bias terms; r is the scaling factor that determines the compression ratio of the channels; and σ represents the Sigmoid activation function.

#### 2.1.4. FusedMBConv

**Figure 6:**
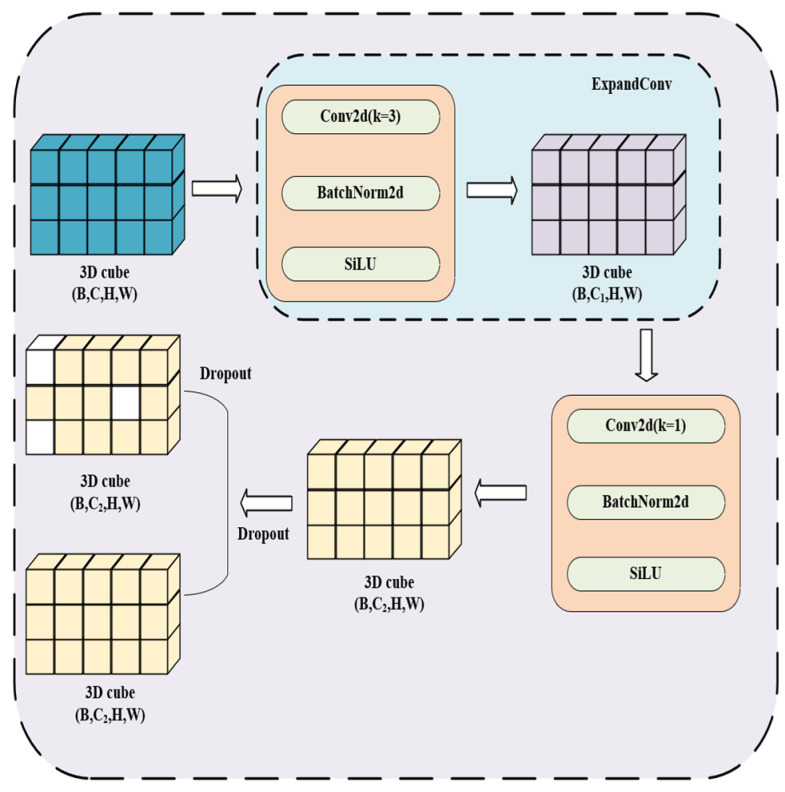
Structural diagram of the FusedMBConv module. This module optimizes computational efficiency by merging the expansion and depthwise convolutions into a single 3 × 3 convolution. The diagram illustrates the streamlined process of simultaneous channel expansion and spatial feature extraction, followed by a 1 × 1 projection and dropout for improved inference speed. The arrows in the figure indicate the direction of data and feature flow through each computational layer. Different colored 3D cubes represent feature maps at distinct processing stages: the blue cube denotes the input feature map, the purple cube denotes the feature map after the ExpandConv layer, and the yellow cubes denote intermediate and output feature maps after 1 × 1 projection. The white blocks in the yellow feature maps represent units randomly zeroed by the dropout operation to prevent overfitting.

The FusedMBConv module combines the first two convolutions of the MBConv-TCSA module into a single standard 3 × 3 convolution, directly achieving both channel expansion and spatial feature extraction, as illustrated in Figure 6. Specifically, the design of the FusedMBConv module can be broken down into the following steps:

The input tensor $x\;\epsilon R^{C*H*W}$ is processed by the module, where C represents the number of input channels, and H and W denote the height and width of the image, respectively. Based on this, the input undergoes channel expansion through an expansion convolution operation, which is defined as follows:

(14) $$ExpandOutput=\sigma \left(BN\left({Conv}_{3\times 3}\left(x\right)\right)\right){Conv}_{3\times 3}:R^{c}\to R^{e}(e=c\times r)$$

where ${Conv}_{3*3}$ refers to a convolution operation with a 3 × 3 kernel, σ is the SiLU activation function, and e denotes the expansion factor. This process can be viewed as expanding the feature map through a convolution layer to a higher number of channels, thus enhancing the representational capacity of the feature map.

Next, the output from the expansion convolution is processed through a projection convolution to map the channel dimension to the final output channel size. This process is represented as:

(15) $$ProjectOutput=\;\sigma \left(BN\left({Conv}_{1\times 1}\left(ExpandOutput\right)\right)\right){Conv}_{1\times 1}:R^{e}\to R^{C_{out}}$$

where ${Conv}_{1*1}$ refers to a convolution operation with a 1 × 1 kernel, BN denotes batch normalization, and σ is the SiLU activation function. To further improve the generalization ability of the model and prevent overfitting, a dropout operation is also introduced at the end of the module.

The FusedMBConv module optimizes computational efficiency by merging the expansion and projection convolutions, thus eliminating redundant convolution operations. This design significantly enhances inference speed and conserves computational resources, which is critical for real-time applications.

#### 2.1.5. TCSA-Efficientnet

The TCSA-Efficientnet consists of the following components:

1. Base Layer

In the base layer, a 3 × 3 convolution operation is applied to expand the input image’s channel count from 3 to 24. Additionally, a downsampling operation with a stride of 2 is employed to reduce the spatial resolution of the input. This process can be seen as an initial stage of processing, where fundamental feature information is extracted from the image.

2. Backbone Layer

**Figure 7:**
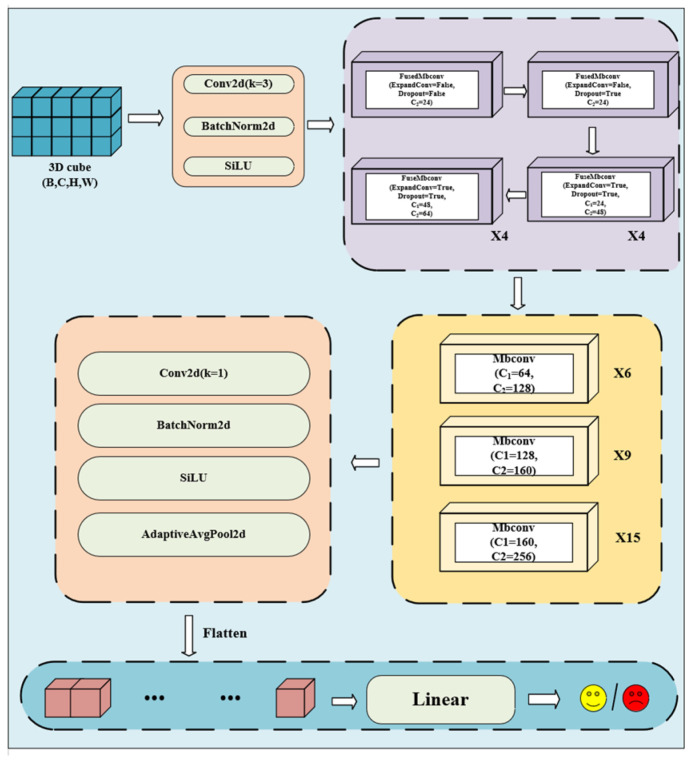
Overall architecture of the TCSA-Efficientnet. The model is organized into three main stages: (1) a Base Layer for initial feature extraction; (2) a Backbone Layer consisting of stacked FusedMBConv and MBConv-TCSA modules for deep spatiotemporal learning (as detailed in Algorithm 1); and (3) a Head Layer for global feature aggregation and final emotion classification. The specific repetition counts and expansion ratios for each block are optimized to balance model depth and computational cost. The arrows in the figure indicate the direction of data and feature flow through each computational layer of the network. The yellow smiley face represents positive emotion states (e.g., high valence/high arousal), and the red frowny face represents negative emotion states (e.g., low valence/low arousal), corresponding to the final emotion classification results output by the model.

The backbone layer serves as the core of the model, primarily composed of multiple MBConv-TCSA and FusedMBConv modules. Figure 7 and Algorithm 1 illustrate the specific details of the backbone layer, including the stacking sequence of these modules and their detailed parameter configurations (e.g., the number of repetitions for each module, expansion ratio, and convolution kernel size). These modules are stacked and configured with varying parameters to facilitate deep feature learning. Each module is repeated multiple times to enhance the model’s expressive power. During this phase, the model progressively abstracts higher-level feature information, which improves its ability to recognize different categories of images.

3. Head Layer

In the head layer, a 1 × 1 convolutional layer is first applied to map the feature map’s channel dimensions to 1280. Then, global average pooling is performed to compress the spatial dimensions to 1 × 1. The feature map is subsequently flattened, followed by a fully connected layer that outputs the classification result. The entire neural network is optimized using a cross-entropy loss function:

(16) $$L=-\frac{1}{N}\sum_{1}^{N}\left[y^{\left(n\right)}\log{\hat{y}}^{\left(n\right)}+(1-y^{\left(n\right)}\log\left(1-{\hat{y}}^{\left(n\right)}\right))\right]$$

where N denotes the batch size, $y^{\left(n\right)}$ ∈ {0, 1} represents the true label of the sample, and ${\hat{y}}^{\left(n\right)}$ is the predicted probability of the model.
**Algorithm 1** Process of TCSA-Efficientnet1: Input: x: (B, C, H, W) 2: x ← ConvBNAct #Start TCSA-Efficientnet blocks 3: for i in range (2): x ← FusedMBConv 4: for i in range (4): x ← FusedMBConv 5: for i in range (4): x ← FusedMBConv 6: for i in range (6): x ←MBConv-TCSA 7: for i in range (9): x ← MBConv-TCSA 8: for i in range (15): x ← MBConv-TCSA # End TCSA-Efficientnet blocks 9: x ← AdaptiveAvgPool2d (1) 10: x ← Flatten (x) 11: x ← Linear (num_features, num_classes) 12: Output: y: (B, num_classes)

### 2.2. Experiment

#### 2.2.1. Dataset Introduction

1. DEAP

**Figure 8:**
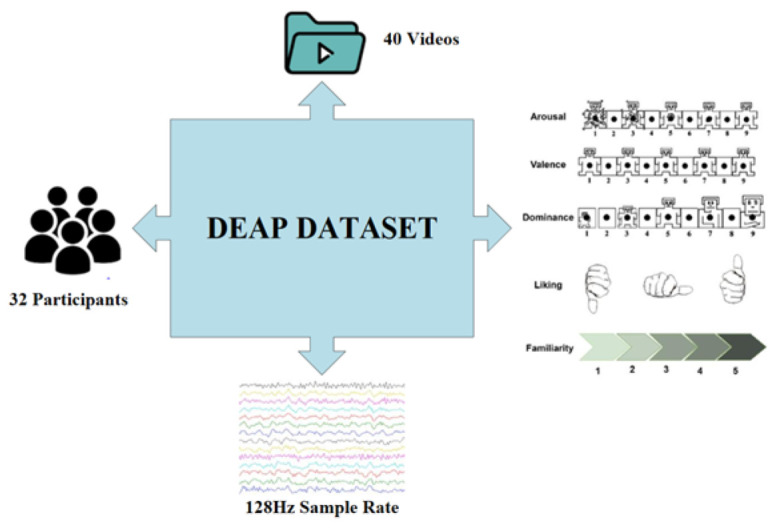
DEAP dataset collection process. The colored lines represent multi-channel EEG signals recorded at a 128 Hz sampling rate during the experiment, with each color corresponding to a distinct EEG electrode channel. The numbers 1–9 in the electrode placement diagrams denote the indices of the EEG recording electrodes, corresponding to the standard International 10–20 system channel positions. The hand gesture symbols represent the participant self-assessment scales for emotional states: the left/right/thumb-up gestures correspond to valence, arousal, and dominance ratings, respectively, used to label the emotional states in the DEAP dataset.

The DEAP (Database for Emotion Analysis using Physiological signals) [9] is a multimodal dataset primarily used in the field of emotion computation, aiming to study human emotional states through the analysis of physiological signals. The experimental setup and data collection process are illustrated in Figure 8. As shown in Table 1, The dataset consists of EEG and peripheral physiological signals collected from 32 participants, who recorded their physiological responses while watching 40 one-minute music video clips. The dataset is designed for emotion classification using video, EEG signals, and physiological signals, and employs decision fusion across different modalities to improve the accuracy of emotion recognition.

2. DREAMER

The DREAMER [25] is a multimodal dataset that records EEG and ECG signals generated during emotional stimulation through audiovisual stimuli. As detailed in Table 2, the ECG data consists of two leads, while the EEG data includes 14 leads. In the dataset, 23 volunteers watched 18 carefully selected and evaluated movie clips. During this experiment, the DREAMER database includes both the participants’ ratings and physiological recordings. Electroencephalogram and electrocardiogram signals were recorded, and each participant rated their emotional states in terms of arousal, valence, and dominance using a five-point scale.

#### 2.2.2. Exchange Channels

In the DEAP dataset, only the first 32 EEG channels are retained. Similarly, in the DREAMER dataset, only 14 EEG channels are retained, and the 2 ECG channels are removed.

In order to enhance the model’s ability to understand the spatial information of EEG signals, the channels were reordered according to the locations of different brain functional areas. The specific order is shown in Table 3 and Table 4:

#### 2.2.3. Experiment Details

In the DEAP dataset, after applying a sliding window to the EEG signals of each participant for each video, each video yields 125 samples, resulting in a total of 5000 samples (40 videos × 125 samples). Therefore, the final data dimension for each participant is 5000 × 3 × 224 × 224 (samples × image channels × image size). In the DREAMER dataset, due to the varying lengths of videos for each participant, the captured EEG samples also differ. The final dimensions for each participant are 6830 × 3 × 224 × 224 (samples × image channels × image size). During all experiments, we used two NVIDIA RTX 3090 GPUs to accelerate the training process. Our model is built on a deep learning framework and optimized for classification tasks.

In the experiment, the batch size, learning rate, and number of epochs were set to 32, $1\times 10^{-2}$ and 200, respectively.

#### 2.2.4. Evaluating Indicator

1. Accuracy

Accuracy measures the proportion of correctly predicted samples to the total number of samples in the model’s predictions.

(17) $$Accuracy=\frac{TP+TN}{TP+TN+FP+FN}$$

where TP enotes true positives, TN denotes true negatives, FP denotes false positives, and FN denotes false negatives.

2. F1-score

F1-score is the harmonic mean of precision and recall.

(18) $$F1=\frac{2\times Precsion\times Recall}{Precison+Recall}$$

where Precison=TP(TP+FP), Recall=TP(TP+FN).

3. AUC (Area Under Curve)

AUC is obtained by calculating the area under the ROC curve. The ROC curve is plotted with the True Positive Rate (TPR) on the vertical axis and the False Positive Rate (FPR) on the horizontal axis.

(19) $$TPR=\frac{TP}{TP+FN}$$

(20) $$FPR=\frac{FP}{FP+TP}$$

#### 2.2.5. Experiment Design

In our study, all samples from different trials for each subject were reassembled. To evaluate the performance of our method, we employed 10-fold cross-validation. This process involves randomly dividing each subject’s EEG data into 10 equal subsets according to the proportion of binary classification labels, ensuring that the ratio of positive and negative samples in each subset is consistent with the original data. Nine of these subsets are used for training, and the remaining one subset serves as the test set. This process is repeated 10 times, with a different subset used as the test set each time, ensuring that each subset is used as a test set once. The average accuracy of the 10 validation results for each subject is calculated, and the average accuracy across all subjects is used as the final accuracy metric. To demonstrate the effectiveness of this method, we conducted extensive experiments and compared the results with baseline models and the latest EEG emotion classification models.

1. Baseline Models

To further validate the effectiveness of the proposed TCSA-Efficientnet model, we applied common benchmark models for image classification to classify recursive graphs. These methods include Decision Trees (DT), Support Vector Machines (SVM), Multi-Layer Perceptrons (MLP), 3D Convolutional Neural Networks (3DCNN), and Dynamic Graph Convolutional Neural Networks (DGCNN).

DT/SVM/MLP: DT and SVM are traditional machine learning methods commonly used as baseline models. MLP is a widely used deep learning model typically employed for classification tasks.

3DCNN: 3DCNN is a deep learning model specifically designed for processing spatiotemporal data, effectively capturing spatiotemporal features within time-series data.

DGCNN: DGCNN is a deep learning model specialized for processing graph data or irregular data.

The aforementioned baseline methods and results are adapted from [26]. Although the preprocessing of raw EEG data in these baseline methods differs, baseline removal and 10-fold cross-validation were performed to divide the training and test sets, enhancing the fairness of the comparative experiments and the reliability of the experimental results.

2. State-of-the-art Models

The proposed TCSA-Efficientnet model was compared with several state-of-the-art and representative EEG-based emotion recognition methods from various countries.

CGRU-MDGN simultaneously learned temporal information while extracting local spatial features of EEG signals, and captured non-Euclidean spatial features between EEG signal channels. GANSER combined adversarial training with self-supervised learning for EEG emotion recognition, generating high-quality, diverse simulated EEG samples. MASA-TCN utilized the spatial learning capabilities of Temporal Convolutional Networks (TCN) for EEG emotion regression and classification tasks, introducing a spatially aware temporal layer that enabled the model to adaptively learn the dynamic temporal dependencies within EEG signals. Gompertz Fuzzy Ensemble trained based on fuzzy sets and combined three individual deep learning models. AMDET leveraged a multidimensional global attention mechanism to exploit the complementarity between the spectral-temporal features of EEG data. LResCapsule consisted of a Light-ResNet-based feature extractor and a capsule-based classifier. To address the challenge of small training datasets for EEG signals, a low-parameter Light-ResNet algorithm was proposed to automatically extract deep emotional features from raw EEG signals.

GLFANet utilized the spatial location of EEG signal channels and the frequency-domain features of each channel, constructing an undirected topology graph to represent the spatial connectivity between channels, and subsequently learned deeper features from the undirected topology graph for emotion recognition. DEEP-CCA extended traditional Canonical Correlation Analysis (CCA) from two modalities to multiple modalities, utilizing an attention mechanism to achieve multimodal adaptive fusion by adjusting weight matrices to maximize the generalized correlation between different modalities. CRAM encoded high-resolution EEG signals using convolutional neural networks and applied a convolutional-recurrent attention model to adaptively process EEG signals, thereby enhancing the efficiency of EEG signal analysis. MLF-CapsNet extracted features from raw EEG signals while simultaneously determining emotional states, introducing multi-level feature mapping with different learning layers during the formation of primary capsules, which enhanced the feature representation capability.

DSSA Net constructs a directional graph and combines spatial, spectral and temporal attention to model spatiotemporal-spectral features from EEG signals. DGC-Link uses Chebyshev Linkage, dual-gate and deep network modules to extract regional correlation and multi-channel features for EEG emotion recognition. ATGRNet leverages hierarchical attention, graph convolution with top-k, residual graph readout and TCN to model frequency, channel, spatial and temporal information for EEG emotion recognition.

3. Ablation Experiment

To further validate the effectiveness and structural rationality of the proposed TCSA module, we conduct ablation experiments from two complementary perspectives: Cross-architecture Generalization (to verify the module’s compatibility across different backbones) and Component-wise Ablation Study (to dissect the contribution of each core component within the TCSA module), ensuring a comprehensive and rigorous verification of the module’s design and performance enhancement capability.

First, regarding the Cross-architecture Generalization experiment, the core objective is to verify the generalizability of the TCSA module across diverse neural network architectures. Specifically, we select classic image classification models (CNN, ResNet-18, and Vgg) as baseline backbones, and integrate the TCSA module into each of these models to quantitatively evaluate the performance improvement brought by the module. By comparing the enhancement effect of the TCSA module when embedded in EfficientNet with its performance in the aforementioned traditional models (CNN, ResNet-18, and Vgg), we intend to reveal the variations in the module’s effectiveness across different architectural designs. This comparison not only demonstrates the strong generalizability of the TCSA module but also enables in-depth analysis of its adaptability in high-performance models (e.g., EfficientNet) versus traditional lightweight models (e.g., VGG), thereby providing valuable insights into the module’s practical applicability in real-world affective computing scenarios.

Second, for the Component-wise Ablation Study, we aim to quantify the independent contribution of each core component within the TCSA module and verify the synergistic effect among them. The TCSA module is composed of three indispensable core components: Temporal Convolution, Channel Attention, and Multi-scale Spatial Attention. To systematically explore the role of each individual component and their interactive effects, we design seven experimental groups with the original EfficientNet (without the TCSA module) as the baseline: (1) EfficientNet + Temporal Convolution; (2) EfficientNet + Channel Attention; (3) EfficientNet + Multi-scale Spatial Attention; (4) EfficientNet + Temporal Convolution + Channel Attention; (5) EfficientNet + Temporal Convolution + Multi-scale Spatial Attention; (6) EfficientNet + Channel Attention + Multi-scale Spatial Attention; and (7) EfficientNet + full TCSA module (Temporal Convolution + Channel Attention + Multi-scale Spatial Attention). This Component-wise Ablation Study is specifically designed to clarify the unique functional role of each component, identify the core driver of performance improvement, and validate the rationality of the TCSA module’s integrated structural design by verifying the synergistic enhancement effect among the three core components.

## 3. Results

### 3.1. Experiment Results

**Figure 9:**
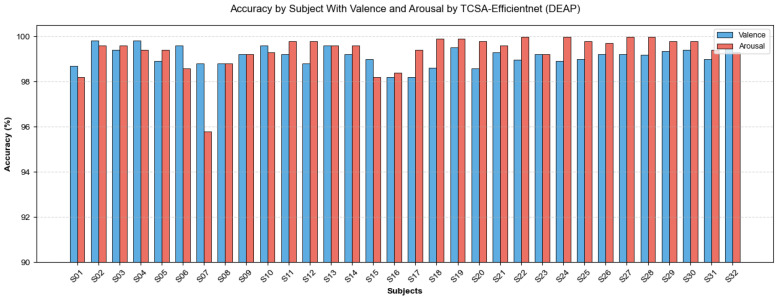
Accuracy of TCSA-Efficientnet for each subject on DEAP.

**Figure 10:**
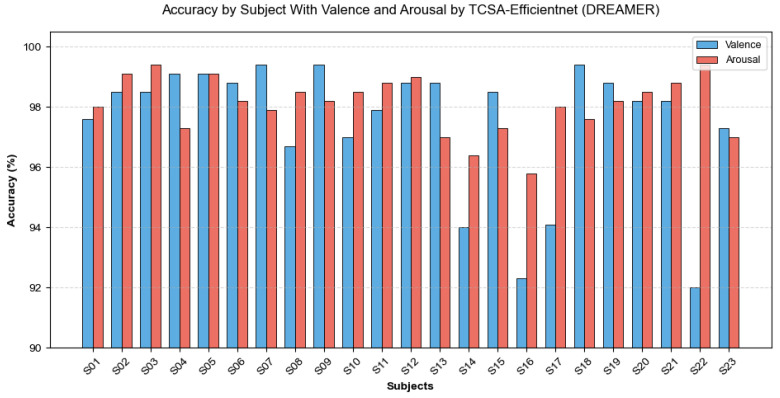
Accuracy of TCSA-Efficientnet for each subject on DREAMER.

As presented in Table 5 and Table 6, the proposed TCSA-Efficientnet model demonstrates its performance on both datasets across the evaluation metrics of Valence and Arousal, reporting accuracy, F1-score, and AUC values. Figure 9 and Figure 10 illustrate the mean accuracy achieved through 10-fold cross-validation for each participant on the respective datasets. On the DEAP dataset, nearly all participants achieved classification accuracies exceeding 95%, with the lowest accuracy observed for Subject 7 in Arousal classification (95.79%). For the DREAMER dataset, all participants attained accuracies above 92%.

### 3.2. Comparsion of DEAP Dataset

As shown in Table 7 of the DEAP dataset, a performance comparison of various methods on the classification tasks is presented, including Accuracy, F1-score, and AUC values. The experimental results demonstrate that the proposed TCSA-Efficientnet exhibits significant superiority in classification performance compared to multiple traditional methods. Specifically, for the two classification tasks, the TCSA-Efficientnet method achieved accuracies of 99.11%/99.33%, F1-scores of 0.98/0.99, and AUC values of 0.99/0.99, surpassing all other comparative methods across these three metrics. Further analysis reveals that TCSA-Efficientnet also demonstrates substantial improvements over traditional machine learning methods such as DT, SVM, and MLP. In terms of accuracy, TCSA-Efficientnet attained improvements of at least approximately 29%, 12%, and 10% compared to DT, SVM, and MLP, respectively, and outperformed 3DCNN and DGCNN by approximately 7%.

Therefore, synthesizing the results from Table 7 indicates that the proposed TCSA-Efficientnet method not only leads in all evaluation metrics but also achieves a substantial advancement in classification performance relative to other methods, demonstrating its superiority on the corresponding dataset.

### 3.3. Comparison of DREAMER Dataset

In the performance comparison for classification tasks in the DREAMER dataset, the proposed TCSA-Efficientnet model demonstrates exceptional performance. As summarized in Table 8, which details the performance of different algorithms on the emotion classification tasks (Valence and Arousal), TCSA-Efficientnet achieved accuracies of 98.08% and 97.49%, F1-scores of 0.97 and 0.92, and AUC values approaching 0.99 and 0.98, respectively. These results surpass those of all other comparative methods. Compared to Decision Trees (DT), Support Vector Machines (SVM), and Multi-Layer Perceptrons (MLP), TCSA-Efficientnet achieved substantial gains in accuracy, outperforming DT, SVM, and MLP by approximately 28, 12, and 11 percentage points, respectively. It also exhibited an advantage of approximately 5 percentage points over 3DCNN and DGCNN. In summary, TCSA-Efficientnet not only leads across all evaluation metrics but also demonstrates substantial improvements in emotion classification tasks on the DREAMER dataset compared to other methods, validating its effectiveness and superiority in processing this type of data.

### 3.4. Comparison of State-of-the-Art Models

Table 9 and Table 10 present detailed comparative results with prior models across different datasets. On the DEAP dataset, the proposed TCSA-Efficientnet model achieves further performance breakthroughs in both classification tasks. Specifically, for the Valence classification task, LresCapsule in lower standard deviations (STD), which decreased from 1.49 and 1.31 to 0.25 and 0.58, respectively, attained an accuracy of 97.45%, while our model elevated this metric to 99.11%, representing an improvement of approximately 1.66 percentage points. For the Arousal classification task, LresCapsule achieved an accuracy of 97.58%, whereas our model reached 99.33%, corresponding to a gain of approximately 1.75 percentage points. Furthermore, our model demonstrated enhanced stability, as reflected in its lower standard deviations (STD), which decreased from 1.49 and 1.31 to 0.25 and 0.58, respectively.

In the DREAMER dataset, the proposed TCSA-Efficientnet model likewise exhibited competitive and superior performance against state-of-the-art methods. As shown in Table 10, for the Valence classification task, the current state-of-the-art DGC-Link model achieved an accuracy of 98.58% (STD = 1.74). Our TCSA-Efficientnet model reached 98.08% accuracy with a much lower STD of 0.93, showing comparable accuracy and stronger stability. For reference, the MLF-CapsNet model yielded an accuracy of 93.94% (STD = 0.37). For the Arousal classification task, DGC-Link attained an accuracy of 92.04% (STD = 5.23), while MLF-CapsNet achieved 94.29% (STD = 0.43). In contrast, our model obtained an accuracy of 97.49% and significantly reduced the STD to 0.21, which surpasses both state-of-the-art approaches in both predictive accuracy and consistency.

This demonstrates that TCSA-Efficientnet not only surpasses the performance of existing state-of-the-art models in emotion classification tasks but also significantly enhances the reliability and stability of the results.

### 3.5. Ablation Experiment

To better validate the enhancement provided by the proposed TCSA module for EEG-based emotion classification tasks, we selected common image classification models, namely CNN, VGG, and ResNet-18, as baselines. In our ablation experiments, the addition of the TCSA module yielded significant performance improvements across all models on classification tasks for both datasets. Detailed results are presented in Table 11 and Table 12.

On the DEAP dataset:

(1) For the CNN model, integrating TCSA resulted in an average accuracy increase of approximately 33.125 percentage points (Valence: +35.28 pp, Arousal: +30.97 pp). Concurrently, the F1-score and AUC improved by approximately 0.69 and 0.43, respectively.
(2) The Vgg model exhibited an average accuracy gain of approximately 33.43 percentage points, with F1-score and AUC improvements of approximately 0.65 and 0.42, respectively.
(3) Although ResNet-18 demonstrated relatively strong baseline performance, incorporating TCSA still led to an average accuracy improvement of 0.9 percentage points, alongside F1-score and AUC gains of approximately 0.01 and 0.002.
(4) Efficientnet achieved an accuracy increase of 0.7 percentage points, with F1-score and AUC improvements of approximately 0.006 and 0.001.

On the DREAMER dataset:

(1) The CNN model showed substantial improvements after TCSA integration: Valence accuracy increased from 63.6% to 75.91% (+12.31 pp), and Arousal accuracy rose from 77.65% to 85.08% (+7.43 pp).
(2) The Vgg model demonstrated a similar trend of improvement: Valence accuracy increased from 63.0% to 79.43% (+16.43 pp), and Arousal accuracy improved from 76.57% to 87.5% (+10.93 pp). This indicates that TCSA provides particularly significant enhancements for models with weaker baseline performance.

Even for initially high-performing models like ResNet-18 and Efficientnet, the TCSA module further optimized results:

(3) ResNet-18 achieved Valence and Arousal accuracy gains of 4.43 and 2.27 percentage points, respectively.
(4) Efficientnet, which exhibited the strongest baseline performance without TCSA, still attained Valence and Arousal accuracy improvements of 2.65 and 2.60 percentage points after TCSA integration.

These findings demonstrate that the TCSA module effectively enhances the emotion classification capability of diverse models. Furthermore, when combined with Efficientnet, TCSA not only increases classification accuracy but also provides additional advantages in terms of stability and efficiency. Therefore, the TCSA module represents a promising solution for achieving high-performance emotion classification.

To further verify the effectiveness of each component in TCSA, a component-wise ablation study is conducted as shown in Table 13. It can be observed that employing only a single component or any pairwise combination results in unbalanced or inferior classification performance on both DEAP and DREAMER, whereas the full TCSA module achieves the highest and most stable accuracy for both Valence and Arousal classification. Although the complete TCSA module has 51.17 M parameters and 5.55 G FLOPs, it enables the three components to work in synergy to fully capture temporal, channel-wise and multi-scale spatial EEG features, eliminating the performance bias and feature incompleteness existing in other variants, and finally delivers the optimal overall emotion recognition performance.

**Figure 11:**
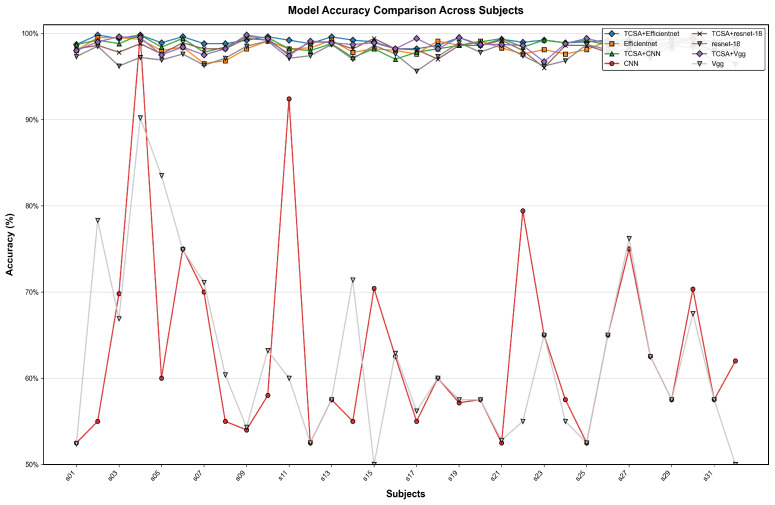
Classification accuracy of TCSA-integrated models on the Valence task of the DEAP dataset.

**Figure 12:**
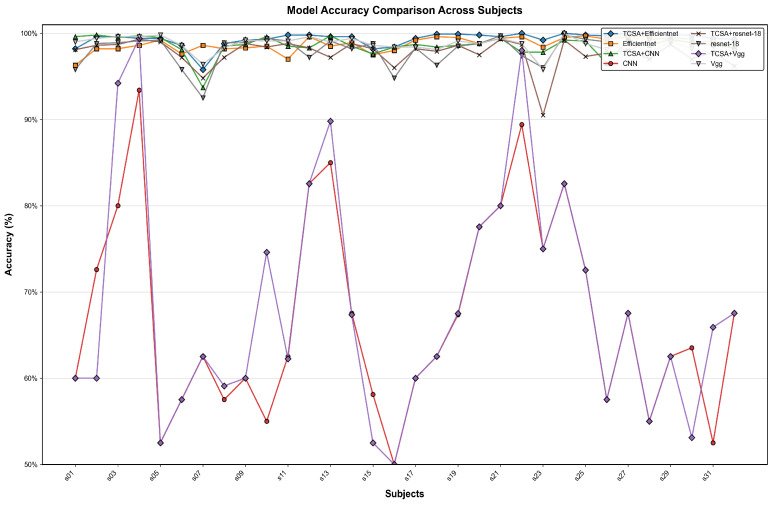
Classification accuracy of TCSA-integrated models on the Arousal task of the DEAP dataset.

**Figure 13:**
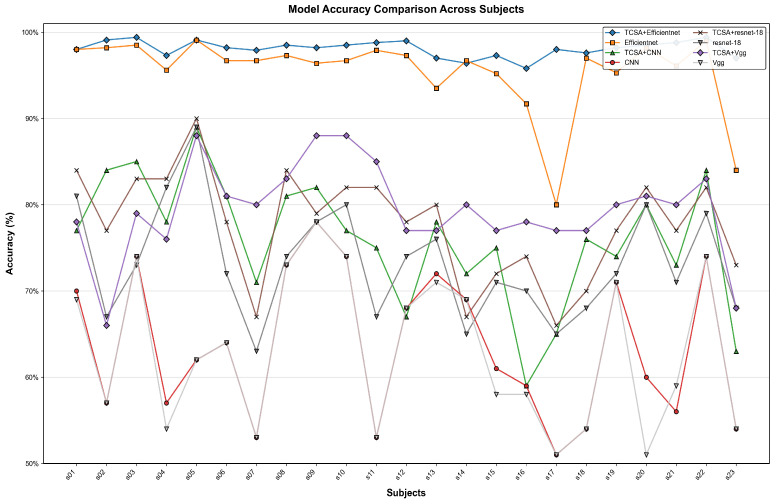
Classification accuracy of TCSA-integrated models on the Valence task of the DREAMER dataset.

**Figure 14:**
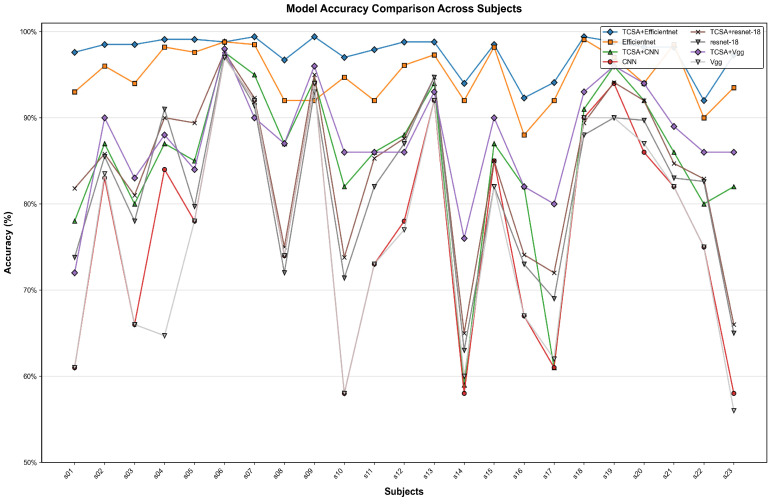
Classification accuracy of TCSA-integrated models on the Arousal task of the DREAMER dataset.

Figure 11, Figure 12, Figure 13 and Figure 14 illustrate the comparative enhancement effects of the TCSA module on the emotion classification performance for each participant across different datasets. The experimental results demonstrate that for models with relatively lower baseline performance, such as CNN and VGG, the integration of the TCSA module led to marked improvements in classification accuracy for every participant. Specifically, within these models, the incorporation of TCSA not only substantially increased the classification accuracy of individual participants but also significantly enhanced the overall model performance. This improvement is attributed to the module’s enhanced capability to capture temporal contextual information within emotional features, thereby enabling more precise emotion classification.

However, for models that already exhibited strong baseline performance, such as ResNet-18 and Efficientnet, while the TCSA module did not yield discernible improvements in classification effectiveness for every single participant, it nevertheless significantly boosted the overall average performance of these models. More importantly, the TCSA module effectively reduced the standard deviation (SD) of the prediction results. This reduction demonstrates the module’s critical role in enhancing model robustness and stability. By minimizing variance, TCSA ensures higher prediction consistency when confronted with diverse data distributions, consequently improving the model’s reliability and generalization capability in practical applications.

**Figure 15:**
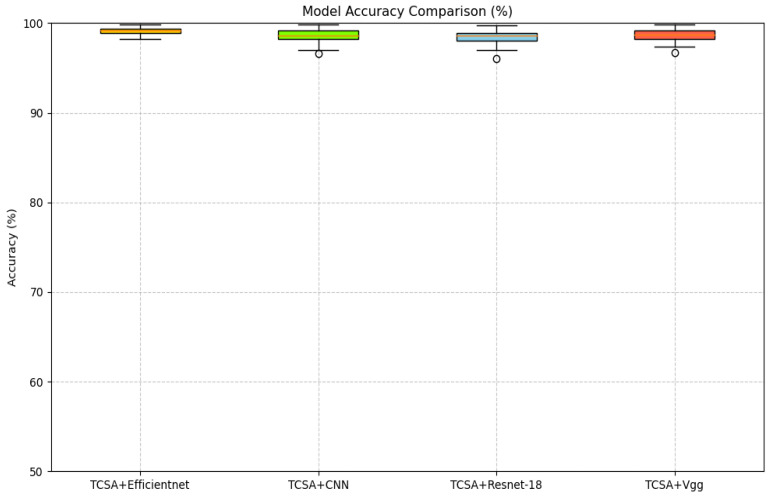
Accuracy comparison for Valence recognition (DEAP) with TCSA.

**Figure 16:**
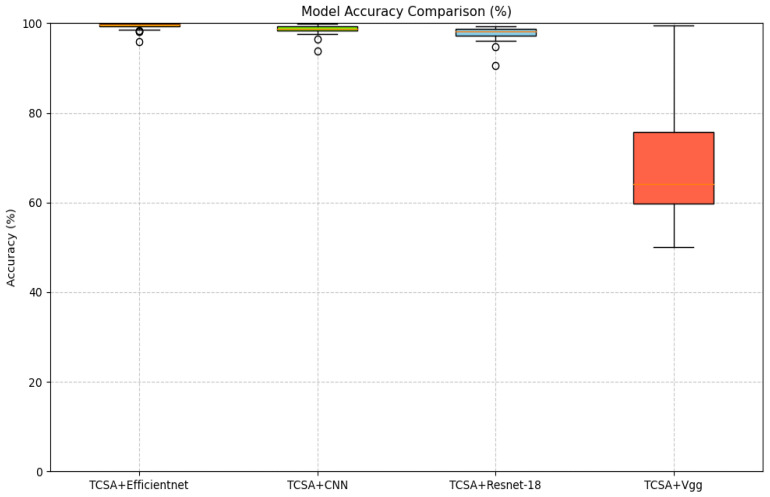
Accuracy comparison for Arousal recognition (DEAP) with TCSA.

**Figure 17:**
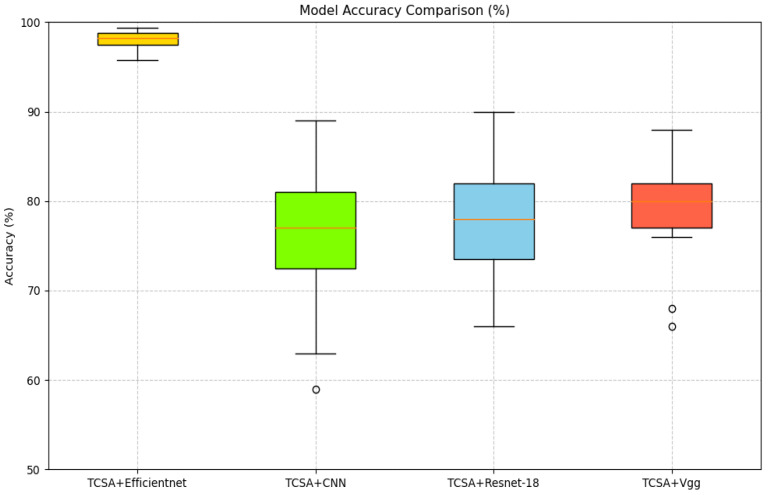
Accuracy comparison for Vanlece recognition (DREAMER) with TCSA.

**Figure 18:**
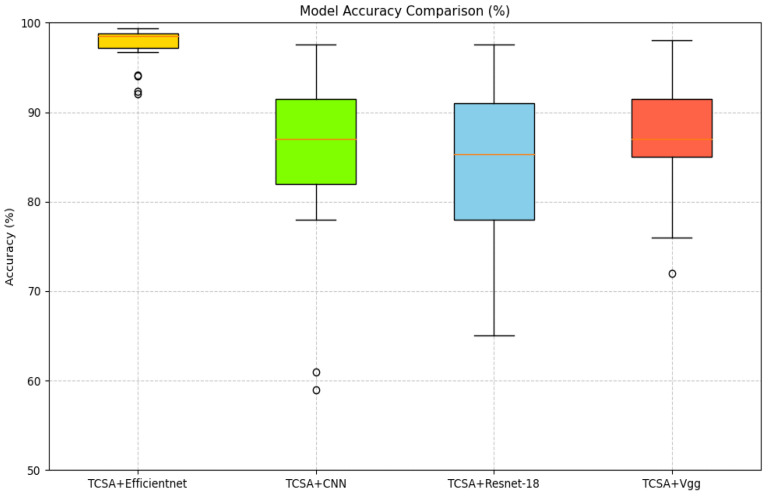
Accuracy comparison for Arousal recognition (DREAMER) with TCSA.

Figure 15, Figure 16, Figure 17 and Figure 18 present a visual comparison of accuracy rates across different classification models augmented with the TCSAmodule. Specifically, TCSA-Efficientnet exhibits minimal fluctuation in prediction accuracy across participants, with a substantially lower standard deviation (STD) compared to other models. This observation indicates the model’s lower sensitivity to individual variations, thereby confirming its stronger robustness.

Furthermore, in terms of average performance, TCSA-Efficientnet attains the highest mean accuracy across all participants, further validating its exceptional generalization capability. This characteristic confers enhanced reliability in practical applications, particularly when confronted with diverse data distributions or complex affective computing scenarios, enabling it to deliver more consistent and precise classification outcomes.

Consequently, when considering both performance and stability comprehensively, TCSA-Efficientnet emerges as the current optimal solution, offering an efficient and robust framework for emotion classification tasks.

## 4. Discussion

The significant performance advantage demonstrated by the TCSA-EfficientNet model in emotion recognition tasks primarily stems from the synergy between spatially informed electrode reordering and nonlinear dynamics modeling. Unlike traditional EEG arrangements that often misalign with the spatial organization of brain structures, our study adopts the International 10–20 system to map physiologically related channels to neighboring pixels. This ensures that the generated EEG images inherently encapsulate the spatial distribution of functional connectivity, enabling the model to more intuitively capture signal correlations between neighboring brain areas. This spatial grounding is further enhanced by the use of Recurrence Plots (RP), which overcome the constraints of conventional frequency-domain feature extraction by preserving the complex temporal and nonlinear characteristics of brain dynamics. When compared with existing state-of-the-art methods in the field, our framework consistently fits at the higher end of accuracy benchmarks. While many current models focus on global graph structures or standard 1D CNNs, our results suggest that transforming 1D time-series into 2D recursive patterns provides a much richer feature set for deep learning backbones like EfficientNet to characterize inter-channel coupling.

Beyond the specific classification performance on benchmark datasets, the robust spatiotemporal feature extraction of this framework opens several possibilities for real-world deployment in smart healthcare and neuro-rehabilitation. For instance, in the mental health field, the proposed model can monitor patients’ emotional changes in real-time, providing doctors with precise data support to assess psychological conditions and adjust treatment plans promptly. Furthermore, for patients with impaired facial or verbal functions, such as those suffering from severe brain injuries or clinical depression, this technology can effectively compensate for the limitations of traditional communication methods by assisting patients in interacting with the healthcare system non-verbally. The TCSA module’s ability to recalibrate feature importance across multiple scales also suggests potential utility in broader applications beyond emotion recognition, such as driver fatigue monitoring or stress detection in high-pressure work environments, where identifying subtle transitions in neural states is paramount for safety and performance.

Despite these advancements, several limitations must be acknowledged to provide a balanced view of the current study. While our results are statistically significant within the subject-dependent framework, the sample size of participants in DEAP and DREAMER remains a constraint for broader population modeling. Although we leveraged data augmentation to ensure enough samples per subject for model convergence, the inherent biological variations—such as individual differences in brain anatomy, scalp conductivity, and baseline brain activity—pose a significant challenge for cross-subject generalization. Moreover, since this research exclusively utilized well-defined, existing benchmarks to ensure reproducibility and fair comparison, the model’s performance on novel subjects in uncontrolled, real-world environments has not yet been fully validated. The potential for “domain shift” caused by different EEG hardware or environmental noise remains a critical barrier for practical deployment. Moving forward, improving robustness across diverse individuals through techniques like domain adaptation, personalized model tuning, or federated learning will be essential to bridge the gap between laboratory success and clinical applicability.

## 5. Conclusions

This study proposes a novel classification framework integrating recurrence plot algorithms with image classification models. Initially, raw multi-channel EEG signals are spatially arranged into topological maps conforming to the International 10–20 system standard, thereby preserving the physiological positional relationships of the electrodes. Subsequently, the one-dimensional time-domain signals from trials are converted into two-dimensional images using the Recurrence Plot (RP) algorithm, where pixel intensities represent local dynamic characteristics of the signals. A specialized module named TCSA is then designed to process these EEG plots. Finally, a modified TCSA-Efficientnet model is employed for classification. This proposed model achieved state-of-the-art classification performance on emotion recognition tasks across two commonly used public EEG emotion datasets.

## Figures and Tables

**Table 1:** DEAP dataset description.

Experiments	Videos	Channels	Sample	Metrics
32	40	40	128 Hz	V/A/D/L

**Table 2:** DREAMER dataset description.

Experiments	Videos	Channels	Sample	Metrics
23	18	16	128 Hz	V/A/D

**Table 3:** The rearranged order of channels in the DEAP dataset and their corresponding cortical regions.

Electrode Channel	Cerebral Cortex Partition
Fp1, Fp2	frontal lobe
AF3, F3, F7, AF4, F4, F8, Fz	Left frontal lobe → right frontal lobe → midline
FC5, C3, T7, FC6, C4, T8, FC1, FC2, Cz	Left central area → Right central area → Median line
CP5, P3, P7, CP6, P4, P8, CP1, CP2, Pz	Left parietal lobe → right parietal lobe → midline
PO3, O1, PO4, O2, Oz	occipital lobe

**Table 4:** The rearranged order of channels in the DREAMER dataset and their corresponding cortical regions.

Electrode Channel	Cerebral Cortex Partition
AF3, F3, F7, AF4, F4, F8	Left frontal lobe → right frontal lobe
FC5, T7, FC6, T8	Left central area → Right central area
P7, P8	Left parietal lobe → right parietal lobe
O1, O2	occipital lobe

**Table 5:** Performance of TCSA-Efficientnet on Valence and Arousal on DEAP. (STD: standard deviation).

	Valence	Arousal
Accuracy/STD	99.11%/0.25	99.33%/0.58
F1-score	0.98	0.99
AUC	0.99	0.99

**Table 6:** Performance of TCSA-Efficientnet on Valence and Arousal on DREAMER (STD: standard deviation).

	Valence	Arousal
Accuracy/STD	98.08%/0.93	97.49%/0.21
F1-score	0.97	0.92
AUC	0.99	0.98

**Table 7:** Average accuracy (%) of two classification tasks on DEAP dataset using different methods.

Models	Valence			Arousal		
	Acc/STD	F1-Score	AUC	Accuracy	F1-Score	AUC
DT [27]	68.28%	-	-	71.16%	-	-
SVM [28]	86.6%	-	-	87.43%	-	-
MLP [27]	87.73%	-	-	88.88%	-	-
3DCNN [29]	89.45%	-	-	90.42%	-	-
DGCNN [30]	92.55%	-	-	93.5%	-	-
TCSA-Efficientnet (ours)	99.11%	0.98	0.99	99.33%	0.99	0.99

**Table 8:** Average accuracy (%) of two classification tasks on DREAMER dataset using different methods.

Models	Valence			Arousal		
	Acc/STD	F1-Score	AUC	Accuracy	F1-Score	AUC
DT [27]	68.28%	-	-	71.16%	-	-
SVM [28]	86.6%	-	-	87.43%	-	-
MLP [27]	87.73%	-	-	88.88%	-	-
3DCNN [29]	89.45%	-	-	90.42%	-	-
DGCNN [30]	92.55%	-	-	93.5%	-	-
TCSA-Efficientnet (ours)	98.08%	0.97	0.99	97.49%	0.92	0.98

**Table 9:** Comparison with state-of-the-art models on DEAP.

Models	Acc%/STD	
	Valence	Arousal
ATGRNet [31]	78.22/18.33	76.46/19.48
CGRU-MDGN [32]	89.45/-	90.24/-
GANSER [33]	93.86/-	94 /-
MT-CNN [34]	96.28/-	96.62/-
Gompertz Fuzzy Ensemble [35]	95.78/-	95.97/-
AMDET [36]	97.48/0.99	96.85/1.66
LresCapsule [26]	97.45/1.49	97.58/1.31
Supernet [37]	94.88/-	93.39/-
GLFANet [38]	94.53/-	94.51/-
DSSA Net [39]	94.97/4.23	94.73/3.27
TCSA-Efficientnet (ours)	99.11/0.25	99.33/0.58

**Table 10:** Comparison with state-of-the-art models on DREAMER.

Models	Acc%/STD	
	Valence	Arousal
Supernet [37]	94.88/-	93.39/-
GLFANet [38]	94.57/-	94.82/-
DEEP-CCA [40]	90.57/-	88.99/-
CRAM [41]	92.27/-	93.03/-
MLF-CapsNet [42]	93.94/0.37	94.29/0.43
DGC-Link [43]	98.58/1.74	92.04/5.23
TCSA-Efficientnet (ours)	98.08/0.93	97.49/0.21

**Table 11:** Ablation study of TCSA on different models on DEAP.

Models	Valence			Arousal		
	Acc	F1-Score	AUC	Acc	F1-Score	AUC
CNN	63.3%	0.2917	0.5633	67.18%	0.3017	0.5834
CNN + TCSA	98.58%	0.9857	0.9987	98.15%	0.9762	0.9970
Vgg	62.41%	0.3379	0.5709	68.4%	0.3571	0.5812
Vgg + TCSA	98.62%	0.9853	0.9985	99.05%	0.9905	0.9991
Resnet-18	97.59%	0.9961	99.6106	97.71%	0.9737	0.9959
Resnet-18 + TCSA	98.41%	0.9840	0.9983	98.69%	0.9856	0.9982
Efficientnet	98.39%	0.9844	0.9985	98.71%	0.9866	0.9979
TCSA-Efficientnet (ours)	99.11%	0.9882	0.9987	99.33%	0.9918	0.9993

**Table 12:** Ablation study of TCSA on different models on DREAMER.

Models	Valence			Arousal		
	Acc	F1-Score	AUC	Acc	F1-Score	AUC
CNN	63.6%	0.2326	0.5217	77.65%	0.09	0.5991
CNN + TCSA	75.91%	0.6521	0.8117	85.08%	0.5658	0.8417
Vgg	63%	0.1804	0.5078	76.57%	0.04	0.566
Vgg + TCSA	79.43%	0.716	0.8508	87.5%	0.6	0.88
Resnet-18	73.26%	0.7752	99.6106	81.83%	0.4886	0.7619
Resnet-18 + TCSA	77.69%	0.6886	0.8239	84.1%	0.5151	0.8015
Efficientnet	95.43%	0.9403	0.9789	94.89%	0.8879	0.959
TCSA-Efficientnet (ours)	98.08%	0.9752	0.991	97.49%	0.925	0.9806

**Table 13:** Component-wise ablation study of TCSA on DEAP and DREAMER.

	DEAP		DREAMER		Parameters	FLOPs
Models	Valence	Arousal	Valence	Arousal		
Efficientnet	98.39%	98.71%	95.43%	94.89%	20.31 M	2.90 G
EfficientNet + Temporal Convolution	98.38%	99.09%	94.39%	96.6%	22.28 M	3.07 G
EfficientNet + Channel Attention	81.58%	99.28%	86.47%	97.23%	20.97 M	2.95 G
EfficientNet + Multi-scale Spatial Attention	99.4%	84%	73.76%	83.43%	48.53 M	5.32 G
EfficientNet + Temporal Convolution + Channel Attention	77.18%	99.02%	94.33%	96.43%	22.94 M	3.12 G
EfficientNet + Temporal Convolution + Multi-scale Spatial Attention	79.22%	79.2%	70.77%	81.29%	50.51 M	5.49 G
EfficientNet + Channel Attention + Multi-scale Spatial Attention	99.13%	99.14%	71.24%	82.73%	49.19 M	5.38 G
EfficientNet + full TCSA module	99.11%	99.33%	98.08%	97.49%	51.17 M	5.55 G

## Data Availability

The data presented in this study are available on request from the corresponding author due to privacy restrictions. The code related to this work has been uploaded to a public GitHub repository: https://github.com/huang0122/EEG-Emotion-Recognition (accessed on 24 March 2026), with the latest stable version v1.0 used for all experiments in this study.
